# Human Coronary Plaque Optical Coherence Tomography Image Repairing, Multilayer Segmentation and Impact on Plaque Stress/Strain Calculations

**DOI:** 10.3390/jfb13040213

**Published:** 2022-11-02

**Authors:** Mengde Huang, Akiko Maehara, Dalin Tang, Jian Zhu, Liang Wang, Rui Lv, Yanwen Zhu, Xiaoguo Zhang, Mitsuaki Matsumura, Lijuan Chen, Genshan Ma, Gary S. Mintz

**Affiliations:** 1 School of Biological Science and Medical Engineering, Southeast University, Nanjing 210096, China; 2 The Cardiovascular Research Foundation, Columbia University, New York, NY 10019, USA; 3 Mathematical Sciences Department, Worcester Polytechnic Institute, Worcester, MA 01609, USA; 4 Department of Cardiology, Zhongda Hospital, Southeast University, Nanjing 210009, China

**Keywords:** coronary, vulnerable plaque, coronary plaque models, multilayer vessel geometry

## Abstract

Coronary vessel layer structure may have a considerable impact on plaque stress/strain calculations. Most current plaque models use single-layer vessel structures due to the lack of available multilayer segmentation techniques. In this paper, an automatic multilayer segmentation and repair method was developed to segment coronary optical coherence tomography (OCT) images to obtain multilayer vessel geometries for biomechanical model construction. Intravascular OCT data were acquired from six patients (one male; mean age: 70.0) using a protocol approved by the local institutional review board with informed consent obtained. A total of 436 OCT slices were selected in this study. Manually segmented data were used as the gold standard for method development and validation. The edge detection method and cubic spline surface fitting were applied to detect and repair the internal elastic membrane (IEM), external elastic membrane (EEM) and adventitia–periadventitia interface (ADV). The mean errors of automatic contours compared to manually segmented contours were 1.40%, 4.34% and 6.97%, respectively. The single-layer mean plaque stress value from lumen was 117.91 kPa, 10.79% lower than that from three-layer models (132.33 kPa). On the adventitia, the single-layer mean plaque stress value was 50.46 kPa, 156.28% higher than that from three-layer models (19.74 kPa). The proposed segmentation technique may have wide applications in vulnerable plaque research.

## 1. Introduction

Intravascular optical coherence tomography (OCT) is a new imaging modality that has been rapidly developing in recent years. It provides unprecedented resolution up to 10 μm, compared to 150–200 μm by intravascular ultrasound (IVUS). It is not only proven to be reliable and widely used in more and more clinical settings [1,2], but also brings advances and breakthroughs to vulnerable plaque research using biomechanics, allowing for more accurate cap thickness quantification and the construction of more realistic models. Nonetheless, the biggest obstacle of OCT is its low penetration depth. That is why some researchers have developed methods to combine IVUS and OCT together to obtain the complete plaque geometry, with IVUS providing the entire vessel wall geometry and OCT providing high-accuracy near-lumen plaque features, especially cap thickness, inflammations and erosion [3,4].

Despite the penetration limitation of OCT, more and more researchers have exploited the abundant information that OCT provides. OCT consensus illustrates the inaccuracy of the statement that OCT has a constant penetration depth [5]. The fact is, OCT penetration depth ranges from 0.1 mm to 2.0 mm and is heavily tissue-dependent. Particularly for high-attenuation tissues such as lipid-rich plaques, light is obscured. For lower-attenuation tissues such as fibrous and calcium, OCT can see deeper structures clearly. A study also showed that more than 180 degrees of external elastic lamina can be recognized in 95% of OCT slices [6]. The enormous visible vessel segments can be taken advantage of to repair the entire vessel wall. 

It is well known that arteries have a three-layer structure: intima, media and adventitia. OCT can actually discriminate the three layers based on image intensity variations [6]. Different layers have different pixel intensities and generate a “light–dark–light” structure in OCT images. It would be a considerable advance if a multilayer vessel wall structure could be obtained from OCT images and used in vulnerable plaque research [7,8,9]. Different layers also have different mechanical properties [10,11,12,13]. The multilayer plaque structure will have a significant impact on plaque stress/strain distributions when multilayer models are used for calculation [14]. 

Recent studies have tried different methods to segment the coronary plaque and vessel wall from OCT images. Athanasiou et al. introduced an automatic segmentation method, which is able to segment four different tissue types in coronary plaque OCT images: calcium (CA), lipid tissue (LT), fibrous tissue (FT) and mixed tissue (MT) [15]. They also applied the edge detection algorithm and ellipse fitting to identify the internal elastic membrane and estimate its bounding area. The method was updated to 3D space using a linear elastic spring mesh method to fully segment the diseased segments for the first time [16]. Zahnd et al. used an original front propagation scheme depending on grayscale gradient information to segment intima, media and adventitia simultaneously in healthy vessel segments [17]. Kafieh et al. introduced a method using a coarse-grained diffusion map for the layer segmentation of retinal OCT images, which has shown robustness even in low contrast and poor layer-to-layer gradient images [18]. Other researchers also put effort into the automatic characterization of OCT plaques using artificial intelligence [19,20].

To the best of our knowledge, the above techniques have not been used to repair three-layered vessel walls simultaneously with subsequent multilayer biomechanical model construction. Three-layer vessel wall models are rarely considered in current modeling research due to the lack of usable segmented multilayer vessel image data.

In this paper, an automatic multilayer segmentation and repair method was developed to segment coronary OCT images to obtain multilayer vessel geometries for biomechanical model construction. Manually segmented data were used as the gold standard for automatic segmentation method development and validation. The edge detection method and cubic spline surface fitting were applied to detect and repair the internal elastic membrane (IEM), external elastic membrane (EEM) and adventitia–periadventitia interface (ADV). The segmented and repaired vessel slices were then used to construct 3D thin-slice models to demonstrate the impact of the multilayer vessel structure on the plaque stress/strain calculation. 

## 2. Materials and Methods

### 2.1. Data Acquisition and Processing

Five existing de-identified intravascular optical coherence tomography (OCT) data sets for patients (*n* = 5) with coronary heart diseases were obtained from Cardiovascular Research Foundation (CRF). One additional patient OCT data set was acquired from Southeast University Affiliated Zhongda Hospital using protocol approved by Southeast University Zhongda Hospital Institutional Review Board (approval code 2019ZDKYSB046) with informed consent obtained. A total of 436 OCT slices from 6 patients (1 male; mean age: 70.0) were used in this study, with demographic data shown in Table 1. OCT images were acquired with ILUMIEN OPTIS System and Dragonfly JP Imaging Catheter (St. Jude Medical, Westford, MA, USA). The spatial resolution of the acquired OCT images was 4.5 μm. Slices with poor image quality were removed from this research. 

### 2.2. Multilayer Automatic Segmentation

**Figure 1:**
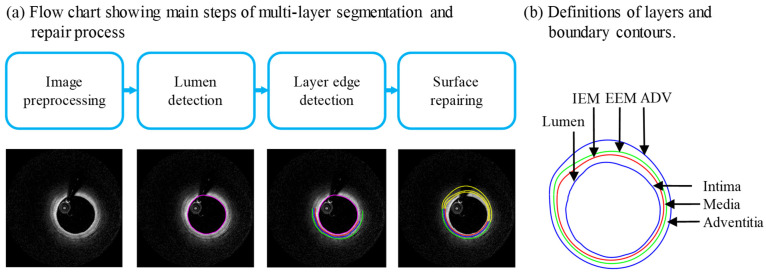
(**a**) Flow chart showing main steps of automatic multilayer segmentation and repair; (**b**) a sample slice showing definitions of lumen, three layers and three boundary contours.

Multilayer vessel wall manual segmentation was performed by trained experts using ImageJ 1.52v software, and served as the gold standard for automatic segmentation method development and validation. Figure 1a gives a flow chart showing the main steps of multilayer automatic segmentation and surface-repairing process using codes based on MATLAB (MATLAB R2021a, MathWorks, Natick, MA, USA). A sample slice showing definitions of lumen, three layers and three boundary contours was given. Details can also be split into three parts as follows.

Part A. Image preprocessing: Intensities of original OCT images were adjusted by changing the window width to increase the contrast of three layers so that intensity gradients between layers were increased and layer boundaries could be better identified. Guidewire artifacts were removed following the method in [16]. Images of one pullback were stacked in polar coordinates to prepare for subsequent segmentations.

**Figure 2:**
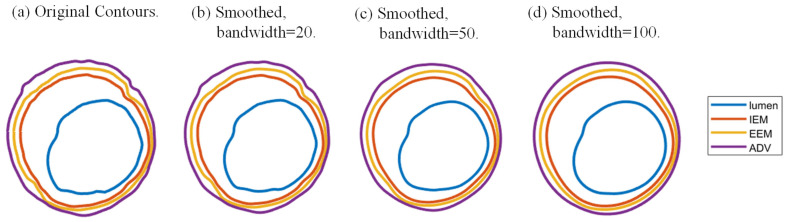
Contours of a sample slice using moving average smoothing with smoothing bandwidths of 20, 50 and 100 pixels.

Part B. Lumen detection: Lumen was detected using Otsu’s thresholding method in each slice [21]. The threshold was given by maximizing between-class variance and minimizing in-class variance, and was then used in the binary classification of vessel tissues and lumen. Image morphological manipulations, using a square structure element whose width was 4 pixels, were performed successively to erase small noises and jump points in the image. Plaque OCT images are often spotty due to irregular plaque morphologies and scattered plaque tissue components and noises and jump points caused by guidewire attachment or residual blood. Small spotty pieces should be removed so that the segmented contours can be used for biomechanical model constructions. Contour smoothing was performed for all slices using a moving average method with a bandwidth of 50 pixels. Figure 2 shows the original and smoothed contours of a sample slice with bandwidths of 20, 50 and 100 pixels. The figure shows that 20 pixels were not enough and that smoothing using 50 pixels was sufficient for modeling use. 

**Figure 3:**
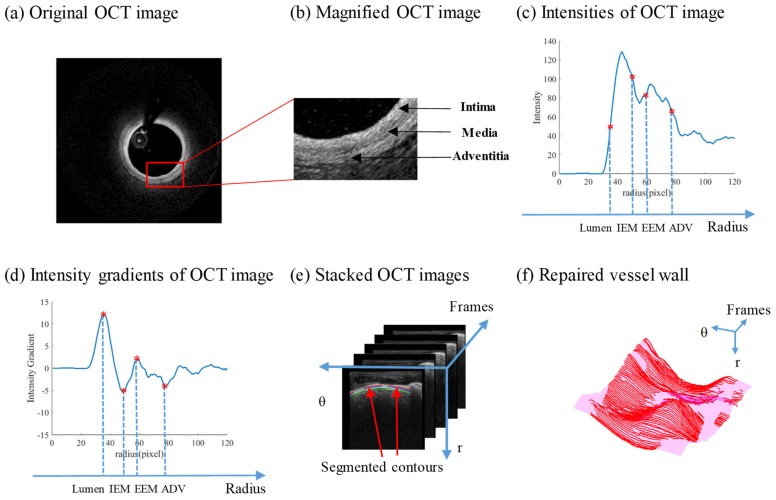
Schematic diagram of OCT multilayer segmentation and repairing. (**a**) Original OCT image; (**b**) magnified OCT image. A “light–dark–light” three-layer structure can be clearly seen; (**c**) radial intensities of OCT image; (**d**) radial intensity gradient of OCT image. Gradient reaches its peak or valley at layer boundary; (**e**) OCT images and the segmented contours were stacked in polar coordinates; (**f**) the whole vessel wall was repaired using known contour segments. Red: contour segments. Magenta: repaired surface.

Part C. Layer edge detection: The graphical basis of detecting different vessel layers is that intima, media and adventitia have different optical properties and pixel intensities in OCT images, and image intensities change most dramatically at layer boundaries. Figure 3a,b show the “light–dark–light” three-layer structure in a healthy vessel slice. Figure 3c,d demonstrate that intensity and intensity gradient had clear patterns in radial direction. From lumen to vessel out-boundary, intensity followed a trend of going up and down twice. Intensity gradient had a similar trend, forming two peaks and two valleys, each representing the boundary between lumen and intima, intima and media, media and adventitia, adventitia and other peripheral tissues. The boundary between intima and media is called internal elastic membrane (IEM), which is a thin membrane mainly composed of elastin. The boundary between media and adventitia is called external elastic membrane (EEM). The boundary between adventitia and other peripheral tissues is called adventitia–periadventitia interface (ADV).

The four boundaries were identified by edge detection based on Canny method, which searches out true edges in large gradient positions amongst large noise [22]. All OCT images were firstly flattened relative to lumen to better align pixels in the same radial depth and increase the efficiency of edge detection [16]. Then, the double thresholds of Canny method and the radius of Gaussian smoothing were continually adjusted by a grid search program to determine the optimal parameters for edge detection. IEM and ADV were detected by the valleys of first derivative; EEM was detected by the peak of first derivative. The whole three-layer structure, as well as the middle of media, were detected by the peak of second derivative, implying a rough candidate region to detect edges. Of all the detected edges, manual selection was performed in the first slice of a pullback to choose the true (seed) edges, and the rest were considered noise edges. Under the assumption that edges between continuous slices do not change dramatically, edges close enough to the seed edges radially in the next slice were specified as the true edges. The remaining slices were performed in the same manner until layers of the whole pullback were segmented.

### 2.3. Surface Repairing

The segmented contours were reverse-flattened first relative to lumen [16]. In 3D polar coordinate space, contours of three layers were stacked and formed three surfaces with holes, as Figure 3e,f showed. Detected contours represent visible vessel inner wall, while the holes represent the invisible parts. The surfaces were then repaired by cubic spline-fitting method to obtain the parts of the vessel wall missing in OCT image [23]. 

After the repair, the complete surfaces were smoothed and transformed from polar coordinate system to Cartesian coordinate system. Contours of three layers are now complete in all the slices, including those obscured by lipid or other tissues.

### 2.4. Multilayer 3D Thin-Slice Models

**Figure 4:**
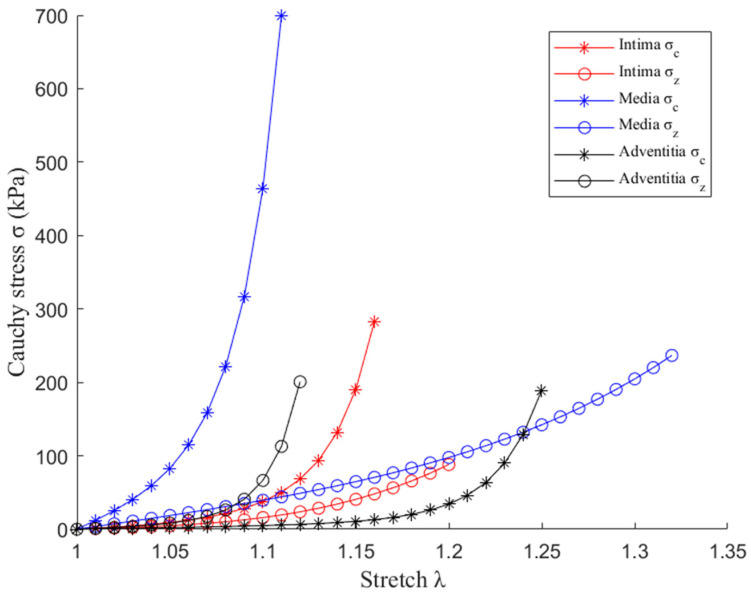
Stress–stretch curves of three layers obtained from uniaxial mechanical testing. σ_c_: Circumferential stress; σ_z_: axial stress. References: intima [24], media [13] and adventitia [11].

Three-dimensional (3D) thin-slice models were constructed for 10 selected slices from one patient using automatically segmented slices obtained from our programs. Both multilayer and single-layer models were constructed to show plaque stress/strain results and the impact of three-layer segmentation on plaque stress/strain calculations. Since OCT data were acquired under in vivo conditions when the vessel was axially stretched and under in vivo pressure, a 5% axial shrink–stretch and a circumferential pre-shrink process were performed to obtain in vivo slice morphology [4]. Vessel tissues were assumed to be hyperelastic, anisotropic, nearly incompressible, and homogeneous. Lipid core was assumed to be hyperelastic, isotropic and nearly incompressible. Modified Mooney–Rivlin material models were used to describe the material properties of vessel tissues, including isotropic and anisotropic parts. The strain–energy density functions for tissue material properties are given below:

(1) $$W_{iso}=c_{1}\left(I_{1}-3\right)+c_{2}\left(I_{2}-3\right)+D_{1}\left[exp\left(D_{2}\left(I_{1}-3\right)\right)-1\right]$$

(2) $$W_{aniso}=W_{iso}+\frac{K_{1}}{K_{2}}\left\{exp\left[K_{2}{\left(I_{4}-1\right)}^{2}\right]-1\right\}$$

where $\;I_{1}=\sum \left(C_{ii}\right)$, $I_{2}=\frac{1}{2}\left[I_{1}^{2}-C_{ij}C_{ij}\right]$, $I_{1}$ and $I_{2}$ are the first and second invariants of right Cauchy–Green deformation tensor $C=\left[C_{ij}\right]=F^{T}F$,$\;F=\left[F_{ij}\right]=\left[\partial x_{i}/\partial a_{j}\right]$; $\left(x_{i}\right)$ is current positionl $\left(a_{j}\right)$ is original position; $I_{4}=\lambda_{\theta }{}^{2}cos^{2}\varphi +\lambda_{z}{}^{2}sin^{2}\varphi$, where $\lambda_{\theta }$, $\lambda_{z}$ are the principal stretches associated with circumferential and axial direction and φ is the angle between the fiber reinforcement and the circumferential direction in individual layers; $c_{1}$, $c_{2}$, $D_{1}$, $D_{2}$, $K_{1}$ and $K_{2}$ are material parameters. Parameter values in the literature were used in this paper: Lipid: $c_{1}\;$= 0.5 kPa, $c_{2\;}$= 0 kPa, $D_{1}\;$= 0.5 kPa, $D_{2}\;$= 1.5; intima: $c_{1}$ = −262.76 kPa, $c_{2}$ = 22.9 kPa,$\;D_{1}$ = 125.9 kPa, $D_{2}$ = 2.0, $K_{1}$ = 7.19 kPa, $K_{2}$ = 23.5; media: $c_{1}$ = −5 kPa, $c_{2}$ = −20 kPa,$\;D_{1}$ = 20 kPa, $D_{2}$ = 2.8, $K_{1}$ = 168 kPa, $K_{2}$ = 57, φ = 24.9°; adventitia: $c_{1}$ = 6.16 kPa, $c_{2}$ = 0 kPa,$\;D_{1}$ = 0.03 kPa, $D_{2}$ = 30, $K_{1}$ = 10 kPa, $K_{2}$ = 54, φ = 75.3° [11,13,24,25]. Uniaxial axial and circumferential stress–stretch plots for intima [24], media [13] and adventitia [11] are given by Figure 4.

The thin-slice models were solved by a finite-element software ADINA 9.6 (Adina R & D, Watertown, MA, USA) following our established procedures [24]. Because stress/strain are tensors, maximum principal stress and maximum principal strain (called stress and strain from here on, respectively) were chosen as their scale representatives for stress/strain comparisons.

### 2.5. Data Extraction and Analysis

**Figure 5:**
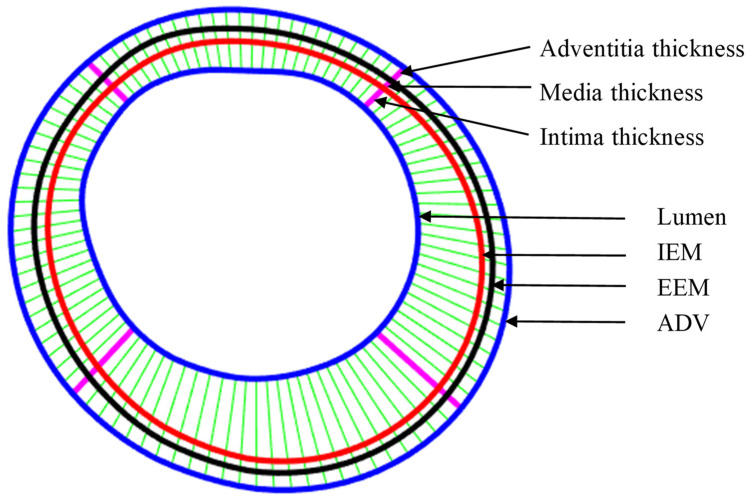
Schematic plot demonstrating the piecewise equal-step method for three-layer thickness and the quarter-dividing method. IEM: intima elastic membrane; EEM: external elastic membrane; ADV: adventitia–periadventitia interface.

Since plaque slices may have irregular and nonuniform geometries, each slice was divided into 4 quarters, with each quarter containing 25 evenly spaced nodal points on the lumen. Each lumen nodal point was connected to a corresponding point on vessel wall using a piecewise equal-step method to deal with irregular nonuniform plaque morphologies [26]. Figure 5 gives an illustration for the definition of layer thickness of the three layers. Specifically, intima thickness was defined as the length of the line segment connecting lumen and IEM. Media and adventitia thicknesses were defined similarly. Layer thickness data were extracted from the 100 nodal points for each slice (total 436 slices from 6 patients) to compare their differences between automatic and manual segmentations. Plaque stress and strain data were also extracted from those nodal points of their corresponding thin-slice models for analysis.

Layer thickness data of the three layers of all slices were stored in matrix $T_{q}\left(i,j,k\right)$, where *q* = 1, 2, 3 represents intima, media and adventitia, respectively; *i* is the point-index for the 100 points in a given slice; *j* is the slice-index for the slices for a given patient; and *k* is the patient index for a given patient (*k* = 1, …, 6). Equation (3) calculates the slice mean thickness of *q*-layer (*q* = 1, 2, 3) of *j*-th slice from Patient *k*. Equation (4) calculates the patient mean thickness of *q*-layer (*q* = 1, 2, 3) of all slices from Patient *k*. Equation (5) calculates the mean thickness of *q*-layer (*q* = 1, 2, 3) for all slices from all patients.

(3) $$\mathrm{Slice}\;\mathrm{mean}\;\mathrm{thickness}\;\mathrm{for}\;q-\mathrm{layer}=\frac{1}{100}\sum_{i=1}^{100}T_{q}\left(i,j,k\right),\;j\;\mathrm{and}\;k\;\mathrm{fixed};$$

(4) $$\mathrm{Patient}\;\mathrm{mean}\;\mathrm{thickness}\;\mathrm{for}\;q-\mathrm{layer}=\frac{1}{100}\times \frac{1}{m}\times \sum_{j=1}^{m}\sum_{i=1}^{100}T_{q}\left(i,j,k\right),\;k\;\mathrm{fixed};$$

(5) $$\mathrm{Mean}\;\mathrm{thickness}\;\mathrm{of}\;q-\mathrm{layer}\;\mathrm{for}\;\mathrm{all}\;\mathrm{patients}=\frac{1}{100}\times \frac{1}{m}\times \frac{1}{n}\times \sum_{k=1}^{n}\sum_{j=1}^{m}\sum_{i=1}^{100}T_{q}\left(i,j,k\right)$$

Thickness error was defined as the relative error between automatic contour thickness, represented by the matrix $T_{q}^{a}\left(i,j,k\right)$, and manual contour thickness, represented by matrix $T_{q}^{m}\left(i,j,k\right).$ Equation (6) calculates the thickness error of *q*-layer ${\mathrm{Error}}_{q}\left(j,k\right)\;$ (*q* = 1, 2, 3) of *j*-th slice of Patient *k*. Equation (7) calculates the thickness error of *q*-layer ${\mathrm{Error}}_{q}\left(k\right)\;$(*q* = 1, 2, 3) of Patient *k*. Equation (8) calculates the thickness error of *q*-layer ${\mathrm{Error}}_{q}$ (*q* = 1, 2, 3) for all patients.

(6) $${\mathrm{Error}}_{q}\left(j,k\right)=\frac{\frac{1}{100}\times \sum_{i=1}^{100}\left({\;T}_{q}^{a}\left(i,j,k\right)-T_{q}^{m}\left(i,j,k\right)\right)}{\frac{1}{100}\times \sum_{i=1}^{100}T_{q}^{m}\left(i,j,k\right)}\times 100\%\;,\;j\;\mathrm{and}\;k\;\mathrm{fixed};$$

(7) $${\mathrm{Error}}_{q}\left(k\right)=\frac{1}{m}\sum_{j=1}^{m}\;{\mathrm{Error}}_{q}\left(j,k\right),\;k\;\mathrm{fixed};$$

(8) $${\mathrm{Error}}_{q}=\frac{1}{n}{\mathrm{Error}}_{q}\left(k\right)$$

## 3. Results

### 3.1. Comparison of Layer Thickness between Automatic and Manual Segmentations

**Figure 6:**
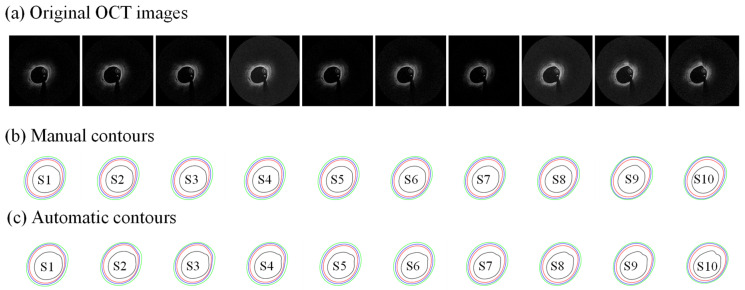
Sample manual and automatic contours derived from OCT images. (**a**) Original OCT images; (**b**) manual contours; (**c**) automatic contours.

Table 2 gives patient thickness values for automatic and manual segmentations from the six patients and errors of automatic segmentations compared to the gold standard (manual segmentation). Errors were calculated using Formulas (6)–(8) with point-to-point differences. Slice-averaged errors with slice standard deviations for each patient are demonstrated in Table 2. Patient-averaged errors with standard deviations are given after P6. Figure 6 gives 10 sample OCT slices selected from one patient, showing their manual and automatic contour differences. It shows that manual and automatic contours were very close. Layer thickness varied greatly from layer to layer and from patient to patient. Intima (which is really the thickened intima, or plaque) had the largest thickness and variance, with an average thickness of 0.6464 ± 0.2222 mm. Intima thickness of P3 (0.8183 mm) was more than twice of that of P4 (0.3963 mm), showing large patient variation. Mean layer thicknesses of media and adventitia were 0.2426 ± 0.0596 mm and 0.2234 ± 0.0587 mm, respectively. Automatic contours derived from our program showed a similar layer thickness compared to manual contours, with 0.6240 ± 0.2174 mm, 0.2290 ± 0.0519 mm and 0.2324 ± 0.0477 mm each for intima, media and adventitia. The relative errors of three layers were mostly less than 10%, with a mean error of −1.40 ± 8.13%, −4.34 ± 11.17% and 6.97 ± 12.00% for intima, media and adventitia, respectively. Negative errors indicated that thicknesses of automatic contours were smaller than those of manual contours. Intima had the smallest error, while media and adventitia had a slightly larger error and variance. This is expected, since accuracy for intima should be better than that for media and adventitia due to OCT penetration limitation. An alternative explanation could be that the absolute placement of the relevant contours is equally accurate/inaccurate for all layers, but because the intima is the thickest layer in these patients, the relative errors (computed by Equation (6)) ended up being smallest. Media thickness tended to be underestimated, while adventitia thickness tended to be overestimated.

Although the mean errors are somewhat informative, they likely obscure local/regional errors that may have been substantially higher (as evidenced, in part, by the standard deviation values). Given that local vessel wall and plaque characteristics are so important for clinical assessment and prognosis, the 90th percentile of the absolute value of the errors for each patient/layer are given by Table 3. The errors were based on slice-averaged results. Pointwise errors could be a little larger.

### 3.2. Point-to-Point Manual and Automatic Contour Distances of Lumen, IEM, EEM and ADV

Table 4 gives distances between corresponding manual and automatic contours (using point-to-point calculation) to show automatic contour locations relative to their corresponding manual contours. Negative values mean that automatic contours had smaller radii than manual contours in polar coordinates. Distance and standard deviation of lumen contours were the smallest, with an average distance of −0.0081 ± 0.0310 mm. The distances of IEM, EEM and ADV contours were −0.0279 ± 0.0539, −0.0689 ± 0.0563 and −0.0153 ± 0.0356 mm, respectively. Table 4 could also lead to a possible explanation for why media and adventitia thickness errors tended to have opposite signs in Table 2. For P1–P3, EEM contours had large negative values, which meant that the automatic method may be consistently placing the EEM contour slightly closer to IEM. For P4, the IEM contour errors were positive and led to a negative media thickness error. 

### 3.3. Impact of Multilayer Segmentation on Plaque Stress/Strain Calculations

Table 5 provided mean stress values of the 10 slices from three-layer and single-layer models and their differences using three-layer values as baseline values. Negative percentage means that the single-layer model provided an underestimate for the stress/strain value(s) calculated. The single-layer mean plaque stress value from lumen was 117.91 ± 5.55 kPa, 10.79% lower than that from the three-layer models (132.33 ± 8.40 kPa). However, on the out-boundary (adventitia), the single-layer mean plaque stress value was 50.46 ± 37.20 kPa, 156.28% higher than that from the three-layer models (19.74 ± 1.78 kPa). 

Table 6 gives mean strain values of the 10 slices from three-layer and single-layer models and their differences. The single-layer mean plaque strain value from lumen was 0.1916 ± 0.0034, 4.88% lower than that from the three-layer models (0.2015 ± 0.0050). On the out-boundary (adventitia), the single-layer mean plaque strain value was 0.1064 ± 0.0058, 13.40% lower than that from three-layer models (0.1228 ± 0.0066). 

**Figure 7:**
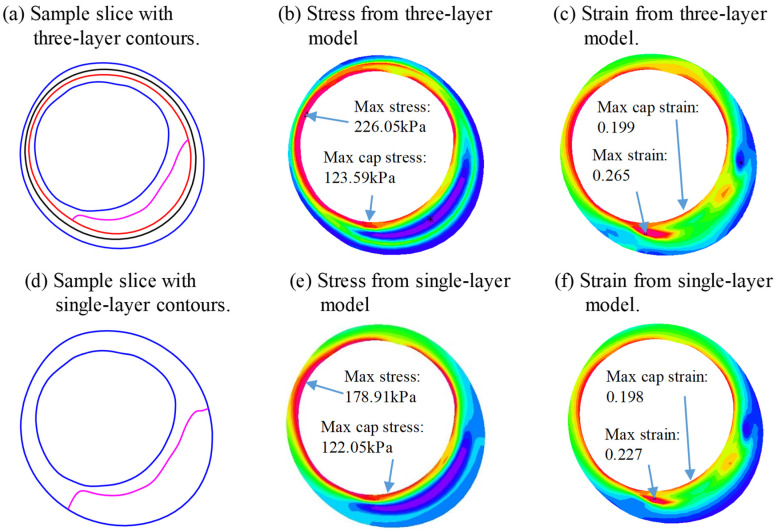
Comparison of plaque stress/strain distributions from three-layer and single-layer models using a sample slice. (**a**) Sample slice with three-layer contours; (**b**) stress from three-layer model; (**c**) strain from three-layer model; (**d**) sample slice with single-layer contours; (**e**) stress from single-layer model; (**f**) strain from single-layer model.

Figure 7 demonstrates the stress/strain distributions from three-layer and single-layer models using a sample slice. It shows that the maximum plaque stress from the three-layer model was 26% higher than that from the single-layer model (226.05 kPa vs. 178.91 kPa), while the maximum strain values from both models were almost identical (0.199 vs. 0.198). The cap stress/strain values did not show much difference.

## 4. Discussion

### 4.1. Multilayer Automatic Coronary Plaque OCT Segmentation, Repairing and Its Significance to Vulnerable Plaque Research

Mechanical forces play an important role in coronary plaque initiation, progression and its eventual rupture, which often leads to critical clinical events such as heart attack and acute coronary syndrome (ACS). Accurate calculation of plaque stress/strain is of vital importance to vulnerable plaque research, including plaque progression prediction, vulnerability assessment, plaque rupture prediction and patient diagnosis, management and treatment plan optimization. Plaque stress/strain calculations depend on plaque morphology, components, pressure conditions and plaque tissue material properties. Most current plaque models use single-layer vessel structures, primarily due to image modality resolution limitations. The development and acceptance of OCT in clinical practice provide the possibility of obtaining multilayer vessel geometries. The multilayer automatic coronary plaque OCT segmentation and repairing technique proposed in this paper make it possible to use three-layer models to improve the accuracy for plaque stress/strain calculations. It will have a considerable impact on plaque progression and vulnerability predictions.

Automation is also one of the contributions of this work. The manual annotation of OCT vessel wall is extremely laborious, especially multilayer annotation. As different layer boundaries are close to each other and are hard to discriminate by naked eyes, it takes huge time and labor cost to annotate multilayers, and also brings in accidental errors. We demonstrated that multilayer thicknesses were precisely quantified using our automated process around the whole vessel wall with relative errors of 1.40%, 4.34% and 6.97% (for intima, media and adventitia, respectively) compared to manual segmented contours (gold standard), respectively. 

### 4.2. Limitations 

Some limitations of this study include: (a) Small patient size—this is a pilot study, and a larger-scale study is needed to further validate the feasibility of this method and bring it into clinical practice. (b) The method is not able to automatically characterize other plaque components such as lipid and calcification. Plaque components were manually annotated in this study. More advanced technologies such as artificial intelligence and neural network should be integrated to achieve entire automatic OCT segmentation. (c) Three-dimensional thin-slice models were used for model construction efficiency. Full 3D models should be used for better accuracy. (d) Tissue material properties used parameter values from the literature since patient-specific material properties were not available. (e) Layered plaques have different layer patterns, which is confusing for edge detection. This is a major error source [27,28,29]. (f) Some artifacts (such as sudden axial removal of OCT guidewire because of heart motion during acquisition) can also lead to spatial discontinuity in sequential slices, resulting in the wrong region to select true edges. (g) The whole analysis process for a patient, including automatic segmentation, repairing and thin-slice modeling, needs about 3–4 h. We will continue to try to shorten the analysis time and get closer to clinical implementations. 

### 4.3. Future Challenges and Directions

More precise and higher-quality coronary plaque images are essential to vessel reconstruction and modeling. With the development of biomedical technologies, more advanced imaging modalities, such as a dual catheter combining both IVUS and OCT, are becoming available, providing better vessel images [30]. In terms of modeling, quantification of vessel material property has always been a pain point, and more accurate and patient-specific material parameters are needed. There is also a balance between model accuracy and model construction cost that we need to keep. Automation of modeling and data processing are needed for clinical implementation. A multilayer fluid–structure interaction (FSI) model is desirable to have and has great potential for studying the biomechanical behavior of different layers considering both solids and fluids. However, labor cost is a big concern. The automation of the 3D thin-slice model can greatly shorten the distance between laboratorial experiments and clinical practice.

## Figures and Tables

**Table 1:** Patient demographic data. BP: blood pressure; RCA: right coronary artery; LCX: left circumflex artery; LAD: left anterior descending artery; HT: hypertension; DM: diabetes mellitus; HL: hyperlipoproteinemia.

Patient	Age	Sex	Vessel Segment	BP (mmHg)	Number of Slices	Comorbidities
P1	80	F	RCA	138/71	75	HT DM
P2	65	F	RCA	149/63	90	DM
P3	74	F	RCA	151/62	76	HT DM HL
P4	62	F	RCA	117/79	75	HL
P5	72	M	LCX	143/80	60	HT DM HL
P6	67	F	LAD	113/60	60	Not available

**Table 2:** Summary of layer thickness values and slice-averaged errors of 6 patients with slice standard deviations.

Patient	Intima (mm)			Media (mm)		
	Auto	Manual	Error	Auto	Manual	Error
P1	0.6298 ± 0.0948	0.6661 ± 0.1009	−4.82 ± 4.30%	0.2585 ± 0.0455	0.2655 ± 0.0335	−5.27 ± 5.20%
P2	0.7262 ± 0.2575	0.7794 ± 0.2346	−4.09 ± 5.71%	0.2662 ± 0.0270	0.2880 ± 0.0203	−7.38± 7.58%
P3	0.7763 ± 0.2151	0.8183 ± 0.1945	−5.04 ± 7.21%	0.2613 ± 0.0215	0.2871 ± 0.0241	−8.76 ± 5.24%
P4	0.4268 ± 0.1478	0.3963 ± 0.1416	9.00 ± 5.11%	0.2146 ± 0.0472	0.2386 ± 0.0565	−9.30 ± 4.11%
P5	0.6439 ± 0.0935	0.6246 ± 0.0989	4.37 ± 5.13%	0.1934 ± 0.0102	0.1845 ± 0.0237	7.03 ± 14.65%
P6	0.4973 ± 0.1740	0.5390 ± 0.1674	−7.25 ± 6.40%	0.1493 ± 0.0082	0.1526 ± 0.0279	1.80 ± 16.93%
Patient-Averaged Mean ± SD	0.6240 ± 0.2174	0.6464 ± 0.2222	−1.40 ± 8.13%	0.2290 ± 0.0519	0.2426 ± 0.0596	−4.34 ± 11.17%
Patient	Adventitia (mm)			Total Vessel (mm)		
	auto	manual	error	auto	manual	error
P1	0.2429 ± 0.0325	0.2151 ± 0.0319	13.49 ± 5.55%	1.1312 ± 0.1227	1.1467 ± 0.1225	−1.32 ± 3.24%
P2	0.2377 ± 0.0451	0.2231 ± 0.0563	8.96 ± 10.87%	1.2301 ± 0.2968	1.2904 ± 0.2713	−5.29 ± 4.55%
P3	0.2217 ± 0.0441	0.2073 ± 0.0429	8.63 ± 11.00%	1.2593 ± 0.2115	1.3127 ± 0.2028	−4.16 ± 3.94%
P4	0.2097 ± 0.0465	0.2037 ± 0.0641	7.76 ± 9.87%	0.8510 ± 0.2310	0.8386 ± 0.2409	−1.91 ± 3.21%
P5	0.2745 ± 0.0402	0.2994 ± 0.0428	−5.64 ± 16.01%	1.1119 ± 0.0857	1.1085 ± 0.1003	0.53 ± 4.70%
P6	0.2112 ± 0.0531	0.2036 ± 0.0531	5.37 ± 9.19%	0.8578 ± 0.1952	0.8952 ± 0.1794	−4.49 ± 5.50%
Patient-Averaged Mean ± SD	0.2324 ± 0.0477	0.2234 ± 0.0587	6.97 ± 12.00%	1.0855 ± 0.2650	1.1124 ± 0.2711	−2.26 ± 5.00%

**Table 3:** The 90th percentile of the absolute thickness errors for 6 patients.

Patients	IEM (mm)	EEM (mm)	ADV (mm)
P1	9.78%	12.84%	18.08%
P2	11.65%	13.16%	20.34%
P3	17.68%	13.19%	20.00%
P4	14.90%	13.06%	19.33%
P5	11.47%	32.58%	29.12%
P6	14.57%	28.42%	16.54%

**Table 4:** Point-to-point manual and automatic contour distances of lumen, IEM, EEM and ADV.

Patient	Lumen (mm)	IEM (mm)	EEM (mm)	ADV (mm)
P1	−0.0037 ± 0.0421	−0.1075 ± 0.0660	−0.1423 ± 0.0790	0.0147 ± 0.0626
P2	0.0140 ± 0.0279	−0.0392 ± 0.0453	−0.1164 ± 0.1021	−0.0690 ± 0.0668
P3	0.0160 ± 0.0312	−0.0259 ± 0.0479	−0.0942 ± 0.0777	−0.0302 ± 0.0449
P4	0.0377 ± 0.0412	0.0613 ± 0.1438	−0.0104 ± 0.1346	0.0117 ± 0.1147
P5	−0.0547 ± 0.0484	−0.0213 ± 0.0360	−0.0123 ± 0.0304	−0.0372 ± 0.0571
P6	0.0177 ± 0.0547	−0.0348 ± 0.0542	−0.0381 ± 0.0655	0.0184 ± 0.0859
Mean ± SD	−0.0081 ± 0.0310	−0.0279 ± 0.0539	−0.0689 ± 0.0563	−0.0153 ± 0.0356

**Table 5:** Summary of stress difference between multilayer and single-layer models.

Slice	Lumen			Out Boundary		
	Multilayer (kPa)	Single-Layer (kPa)	Error	Multilayer (kPa)	Single-Layer (kPa)	Error
1	134.07	115.97	−13.50%	20.49	48.09	134.69%
2	138.01	120.51	−12.68%	21.97	51.38	133.87%
3	114.53	105.99	−7.46%	16.57	42.31	155.33%
4	140.41	121.70	−13.33%	21.91	53.86	145.78%
5	135.68	119.49	−11.93%	20.05	51.69	157.84%
6	134.47	119.16	−11.39%	18.91	50.80	168.59%
7	138.08	122.93	−10.97%	20.17	53.35	164.51%
8	136.50	122.96	−9.92%	20.39	54.05	165.14%
9	131.47	119.76	−8.91%	19.80	52.31	164.16%
10	120.03	110.68	−7.79%	17.13	46.74	172.94%
Mean ± SD	132.33 ± 8.40	117.91 ± 5.55	−10.79 ± 2.20%	19.74 ± 1.78	50.46 ± 37.20	156.28 ± 13.82%

**Table 6:** Summary of strain difference between multilayer and single-layer models.

Slice	Lumen			Out-Boundary		
	Multilayer	Single-Layer	Error	Multilayer	Single-Layer	Error
1	0.2022	0.1895	−6.26%	0.1202	0.1027	−14.56%
2	0.2039	0.1922	−5.76%	0.1248	0.1075	−13.88%
3	0.1903	0.1843	−3.14%	0.1087	0.0941	−13.41%
4	0.2046	0.1925	−5.91%	0.1296	0.1116	−13.86%
5	0.2032	0.1921	−5.46%	0.1267	0.1095	−13.60%
6	0.2036	0.1929	−5.25%	0.1254	0.1087	−13.36%
7	0.2058	0.1954	−5.08%	0.1277	0.1113	−12.83%
8	0.2047	0.1953	−4.58%	0.1272	0.1113	−12.45%
9	0.2019	0.1938	−4.02%	0.1236	0.1078	−12.79%
10	0.1949	0.1883	−3.37%	0.1146	0.0994	−13.24%
Mean ± SD	0.2015 ± 0.0050	0.1916 ± 0.0034	−4.88 ± 1.07%	0.1228 ± 0.0066	0.1064 ± 0.0058	−13.40 ± 0.62%

## Data Availability

Data are available on request. Data cannot be made publicly available for ethical or legal reasons (public availability would compromise patient privacy).
